# A Comprehensive Accounting of Construction Materials in Belt and Road Initiative Projects

**DOI:** 10.1021/acs.est.4c04142

**Published:** 2024-08-20

**Authors:** Lingli Hou, Tomer Fishman, Ranran Wang, Asaf Tzachor, Heming Wang, Peng Wang, Wei-Qiang Chen, Ester van der Voet

**Affiliations:** † Institute of Environmental Sciences (CML), Leiden University, Leiden 2333CC, The Netherlands; ‡ School of Sustainability, Reichman University, Herzliya 4610101, Israel; § Centre for the Study of Existential Risk (CSER), University of Cambridge, Cambridge CB2 1TN, U.K.; ∥ State Environmental Protection Key Laboratory of Eco-Industry, Northeastern University, Shenyang 110819, China; ⊥ Commonwealth Scientific and Industrial Research Organisation (CSIRO), Canberra, ACT 2601, Australia; # Key Lab of Urban Environment and Health, Institute of Urban Environment, Chinese Academy of Sciences, Xiamen, Fujian 361021, China; ∇ University of Chinese Academy of Sciences, Beijing 100049, China

**Keywords:** material stocks, built environment, resource use, infrastructure, sustainability, development initiative

## Abstract

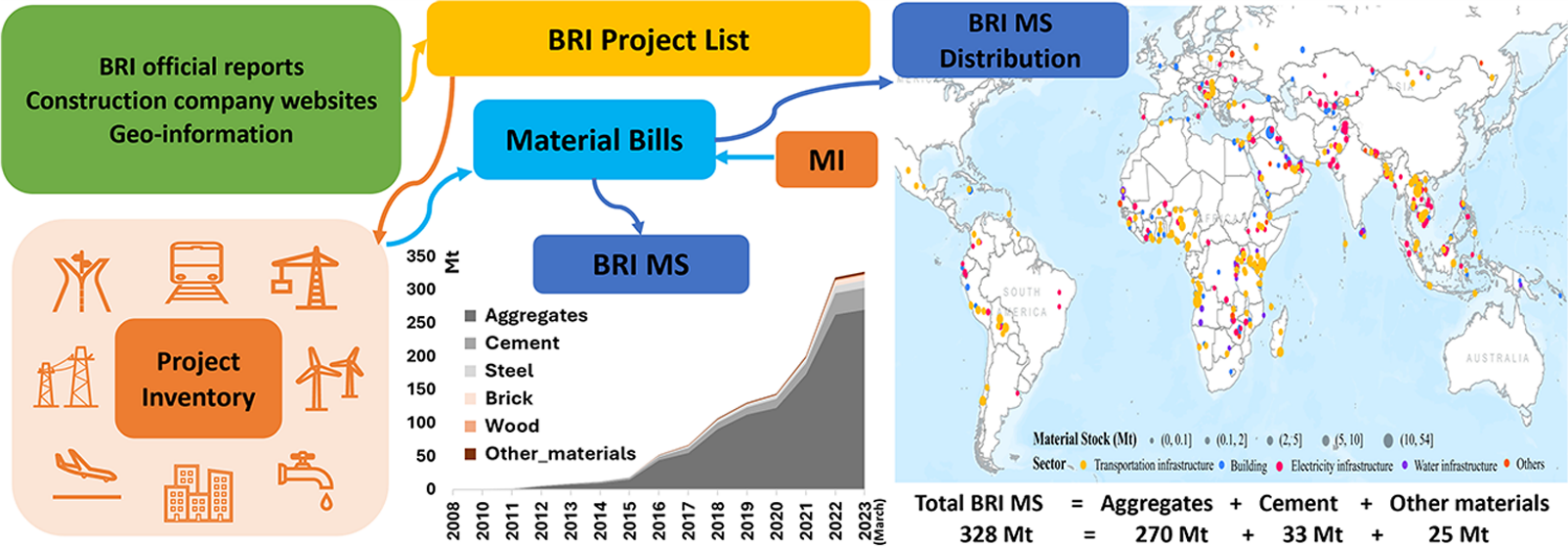

The Belt and Road Initiative (BRI) stands as the most ambitious infrastructure project in history, marked by its scale of investment, extensive geographical reach across continents and countries, and a diverse array of projects from roads to digital networks. While the BRI’s environmental sustainability has raised concerns, the impacts of construction materials used in these projects have been overlooked, especially in developing countries. Here, we map and account for the materials embodied in the BRI by integrating, for the first time, official governmental project reports, geographical information, and material flow analysis. We pinpoint and analyze the BRI material stocks in each individual project by material types, countries, regions, and sectors. Between 2008 and 2023, 328 million tons of construction materials have accumulated in 540 BRI projects around the world, mostly in Asia and Africa. Aggregates (sand and gravel) constitute the largest share (82%), followed by cement, steel, and other materials. Most of the materials are used in transportation infrastructure. Our work further highlights some limitations in terms of data quality for such sustainability assessments. By shedding light on the significant impact of BRI projects on raw material usage across the globe, this study sets the stage for further investigations into environmental impacts of BRI and material stock-flow-nexus from perspective of an initiative.

## Abstract 2

Construction materials have high environmental impacts but are often overlooked, especially in developing countries. This study mapped and estimated the construction material stocks in 540 Belt and Road Initiative (BRI) projects and found ∼328 million tons of construction materials were used by 2023.

## 1 Introduction

The Belt and Road Initiative (BRI), proposed in 2013, is a major global infrastructure and development project undertaken by the Chinese government. It originally set out to include 65 countries, including China, together covering over 60% of the world population and one-third of the world’s trade and GDP.^1−3^ Focused on infrastructure, the BRI’s stated aims are ‘to boost trade, financial integration, and cultural exchanges’.^3−5^ It includes diverse projects like high-speed railroads, electricity networks, multipurposes buildings, and water supply systems.^6,7^ As it entered its tenth year in 2023, the BRI has initiated over 3000 projects–many such as trade, economic, and cultural projects not involving construction–with investments totaling approximately one trillion US dollars.^8^ It has established 200 cooperation agreements with 152 countries and 32 international organizations,^9^ and the numbers continue to grow as the BRI expands.

Despite the BRI’s contribution to regional development,^10−12^ especially in the global south,^5^ there are increasing worries about its sustainability.^13−17^ Researchers have studied the financial aspects of the BRI and have also looked at its country-level and program-wide environmental effects.^18−22^ Not all of the investments made possible by the BRI have been environmentally sound.^23^ The ecological toll of such rapid expansion is profound, as the majority of BRI projects, particularly in transportation, are undertaken with insufficient environmental regulation.^24,25^ Thus, there have been calls for higher global standards in the BRI’s environmental and social governance through thorough strategic evaluations.^13,26^ Research on BRI sustainability has so far been conducted in two approaches: detailed studies of specific countries,^27,28^ and macro-level evaluations across the original 65-country region,^29−32^ revealing a multitude of environmental impacts from carbon emissions to biodiversity loss. Thus, the scope of explorations on the BRI has been markedly broad, with only a handful of studies assessing certain projects individually.^33−36^ Predominantly framed from an economic perspective or as individual case studies, these analyses lack a holistic view, and thus do not address the overarching sustainability of the BRI. Meanwhile, existing BRI projects data sets, though rich in content, are limited—lacking construction details^37−39^ or are not publicly accessible.^40,41^

The development of the BRI has resulted in the accumulation of construction material stocks in massive infrastructure and building projects. Infrastructures enable trade and mobility, contributing to economic development. Globally, construction causes huge inflows of materials in the built environment, as well as huge waste flows,^42,43^ and its contribution to environmental impacts is considerable. The construction sector alone accounts for 23% of global carbon dioxide emissions,^44^ and the production of its materials also generates significant carbon emissions—cement and steel together account for 15% of the world’s carbon emissions.^45,46^ Construction of infrastructure also alters landscapes, affects biodiversity, and produces pollution.^47−49^ Accounting for buildings and structures, containing materials like concrete and steel, is essential for assessing environmental impact side by side with the key roles they play in urbanization and socio-economic advancement.^43,50,51^ Hence, the materials used in BRI projects are not only related to the construction activity itself, but also cause a number of—positive as well as negative—impacts on sustainability, economic, social and environmental aspects. Assessing the mass of material flows and stocks that comprise BRI projects and understanding the resource utilization of constructing them is a critical initial step toward further analysis of the BRI’s sustainability.^43,47^

The objective of this study is to provide details on the raw material usage within the BRI projects, bridging previous gaps in knowledge and data. First, we build our own scope of BRI projects associated with construction material use. Second, we estimate the mass of the major construction materials employed in these BRI projects, which forms a nuanced inventory of material inputs. Finally, we quantify the material stocks in level of projects, countries, regions, and sectors, culminating in a comprehensive database of construction material use of BRI projects around the globe up to March 2023, specifying variations of usage in material types, regional disparities, and sector differences.

## 2 Materials and Methods

**Figure 1:**
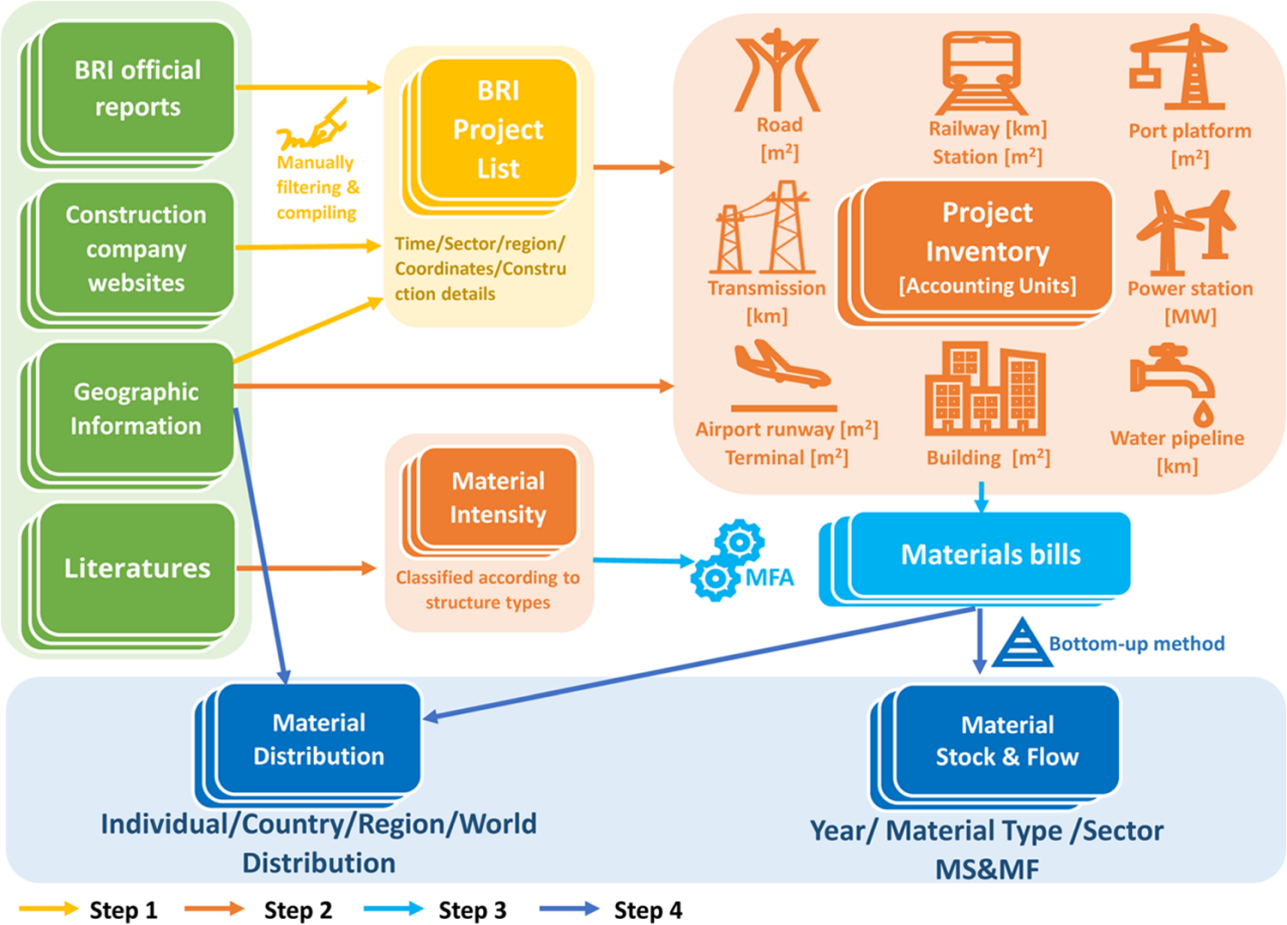
Flowchart for mapping and estimating the material stocks of BRI projects (km: kilometer; m^2^: square meter; MW: Megawatt).

Our approach consists of four steps (Figure 1): (1) compiling a list of BRI projects involving construction; (2) accounting for the size of each project in terms of its units of service, for example, m^2^ floor area and km of rail or pipeline, and investigating the corresponding matching material intensity; (3) estimating the material use in each project (material bills); and (4) calculating material stocks and flows and mapping the distribution of the materials.

### 2.1 Compiling the BRI Projects List

Currently, there is no official list of BRI countries and projects.^25,31,52^ We created a list of 540 relevant BRI projects by gathering data on the name, location, starting year, and (expected) completion year. Using the official Belt and Road Initiative (BRI) Web site as our main source of information,^53^ we included all BRI projects that have been supported by China in various forms, such as financing, design, and construction. Because of the focus on materials that had already been utilized, the projects in our list are either completed or under construction,^53^ excluding those that are agreed on but have not started yet. We referred to information from relevant construction companies’ Web sites,^54−58^ as well as authoritative news Web sites^59^ to complete relevant construction information like starting year and location. We note that although the BRI was officially launched in 2013, some projects that started before are still accounted by China and partner countries as BRI projects in the official Web site.^53^ This means that some of our accounts start before 2013.

We then categorized all projects into 5 sectors: transportation infrastructure, building, electricity infrastructure, water infrastructure, and others (e.g., communication facility and leisure facility). Every project is assigned an ID number, a region based on the geographic information (See Supporting Information SI Table S1), a sector based on the service the project provides, and structure type. We collected data of location of the projects, preferably in terms of coordinates derived from official information. In cases where this information is unavailable, we denoted the location by city or by country. The details of the sectors, project types (e.g., highway, residential building), and structure types are shown in SI Table S2.

### 2.2 Quantifying the Project Service Unit and Matching Material Intensity

Two elements are needed to account for the material stocks in each project:^43,60^ the project inventory measuring each project with suitable accounting unit of service (e.g., buildings measured in m^2^ of floor area, railway measured in km of length, etc.) and matching material intensity (MI) coefficients (e.g., t/m^2^ and t/km, respectively) for each material involved.

#### 2.2.1 Project Inventory

To compile the project inventory, we added to the project list from step one the individual projects’ accounting units of service. Each type of structure type has a different accounting unit, such as length (e.g., km of road), area (e.g., m^2^ for building), and function or service provision (e.g., megawatt of electricity). For projects like railways, power stations, and some of the buildings, the accounting unit can be derived directly from the construction information in the official BRI Web site and construction companies. For some of the roads and buildings, the official sources include no or only partial information, for example lacking the width and number of lanes of road or floor area of buildings. We use two supplementary methods to overcome this gap while ensuring the accuracy of the accounting size. The first one is to integrate Google Maps,^61^ Open Street Maps (OSM),^62^ and site photos from reports and news Web sites. We use these sources to measure the width of the road, width of airport runways, coverage of port platforms, floors and footprint of a building, and so on. However, since many BRI projects are built in recent years, such sources may not yet contain geo-information for all of them. In such cases, we make assumptions on the project: we use other projects in the same category as references, and we compare their features to determine the project size. The detailed steps to decide the accounting units can be found in SI Figure S1. When accounting for train stations and airport terminals we use the material intensities of nonresidential buildings, yet we still categorize their material stocks into the transportation infrastructure sector category due to the service they provided. For water treatment plants we take a similar approach. For each structure type, the total accounting units are listed in SI Table S3.

#### 2.2.2 Material Intensities

We collected archetypal material intensity coefficients for each of the structure types. A total of 14 construction materials are taken into account and grouped to 6 material types: aggregates (sand, gravel, and stones), steel, cement, bricks, wood, and other materials (e.g., plastics, glass, and asphalt). The material intensity coefficients are collected from multiple sources, mostly of material stock accounts and Life Cycle Assessment (LCA) case studies of specific types of construction structures. We prioritized Chinese data as many of the BRI projects are reported as using Chinese construction standards, and added other sources to fill gaps. Table 1 details the material intensity values and their sources. The sources of MI values for bridges, airport runways, and port platforms report concrete as a single material, whose main constituents are cement and aggregates. To be consistent with other structure types, we convert the concrete MIs of these three structures into cement and aggregates by allocating percent weights obtained from previous studies.^63,64^ The original concrete MIs of bridges, airport runways, and port platforms can be found in SI Table S4.

**Table 1:** Material Intensity (MI) Data for BRI Construction Types^a^

structure type	unit	steel	copper	aluminum	wood	cement	aggregates	bitumen	brick	lime	ceramics	glass	plastics	asphalt	refs
railway	t/km	230				160	5340								(65,66)
subway	t/km	439				4383	24,278								(67)
light rail	t/km	359				2917	16,158								
highway	t/m^2^					0.026	1.875	0.012							
secondary road	t/m^2^					0.017	1.381	0.012							
bridge	t/m^2^	0.263			0.518	0.386	2.377							0.146	(68)
airport runways	t/m^2^					0.04	0.671							0.234	(69)
port platforms	t/m^2^					0.048	0.933								
residential	t/m^2^	0.075			0.026	0.238	1.151		0.016	0.027		0.002		0.002	(70)
nonresidential	t/m^2^	0.08			0.027	0.418	1.438		0.234	0.028		0.002		0.002	
thermal power station (<300 MW)	t/MW	87.629	0.925	0.425		165									(71)
thermal power station (300–600 MW)	t/MW	56.701	0.925	0.425		165									
thermal power station (>600 MW)	t/MW	46.392	0.425	0.22		75									
hydropower station (<50 MW)	t/MW	139.175	0.55	0.025		1550									
hydropower station (50–300 MW)	t/MW	221.649	2.9	0.025		950									
hydropower station (>300 MW)	t/MW	77.320	2.9	0.025		280									
wind power station (onshore)	t/MW	134.58	2.8	1.9		53									
wind power station (offshore)	t/MW	136.6	3.4	1.9		153									
PV power station (rooftop)	t/MW	37.113	3	36.5											
PV power station (ground mounted)	t/MW	89.948	5	45.5		45									
nuclear power station	t/MW	48	0.75	0.15		74.5									
transmission	t/km	17		8		4.5									
tap water pipeline	t/km	165				327	3300				3		9.3		(65)
sewer pipeline	t/km	15				327	3300				3		9.3		

a Note: Railways, subways and light rails MIs are accounted as double track rails. The MIs for subway and light rail include auxiliary supporting structures (e.g., tunnels, viaducts, ballast, and sub-ballast). The MI for highway is of expressway asphalt concrete on the ground, and for secondary road it is normal asphalt concrete.

### 2.3 Material Stock Accounting

The material stock of a project is estimated by the following equation
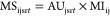
1MS_ijsrt_ is the stock of material i in structure type j, region r, sector s and in year *t*. AU_jsrt_ is the project accounting unit of structure type j in region r, sector s and year *t.* MI_ij_ is the material intensity coefficient for material i in structure type j. For multistructure projects like airports, the material stock is the sum of stock in its composing structures, e.g., the airport runways and terminals.

Considering that these infrastructures have long life span and some of them are still in construction, we assume that material outflows from BRI projects so far are negligible, and do not account for them. Thus, the difference in material stock from year (*t* – 1) to year *t* is treated as the total inflow of materials that occurred in year *t*
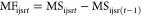
2MF_ijsrt_ is the inflow of material i in structure j, region r, sector s in year *t*. MS_ijsrt_ and MS_ijsr(*t*–1)_ are the stocks of material i in structure j, region r, sector s, in year *t* and year (*t* – 1).

For the total material stock of a sector, we sum the material stocks of the projects in this sector. The equation is as follows
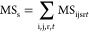
3Combined with the coordinates of projects, we can map the MS of all projects. For the regional analysis, we have grouped the countries with BRI projects into six major world regions, namely East and South Asia, West and Central Asia, Africa, Latin America, Europe, and other regions such as Oceania.

## 3 Results

### 3.1 The Number of BRI Projects Grew Steadily

**Figure 2:**
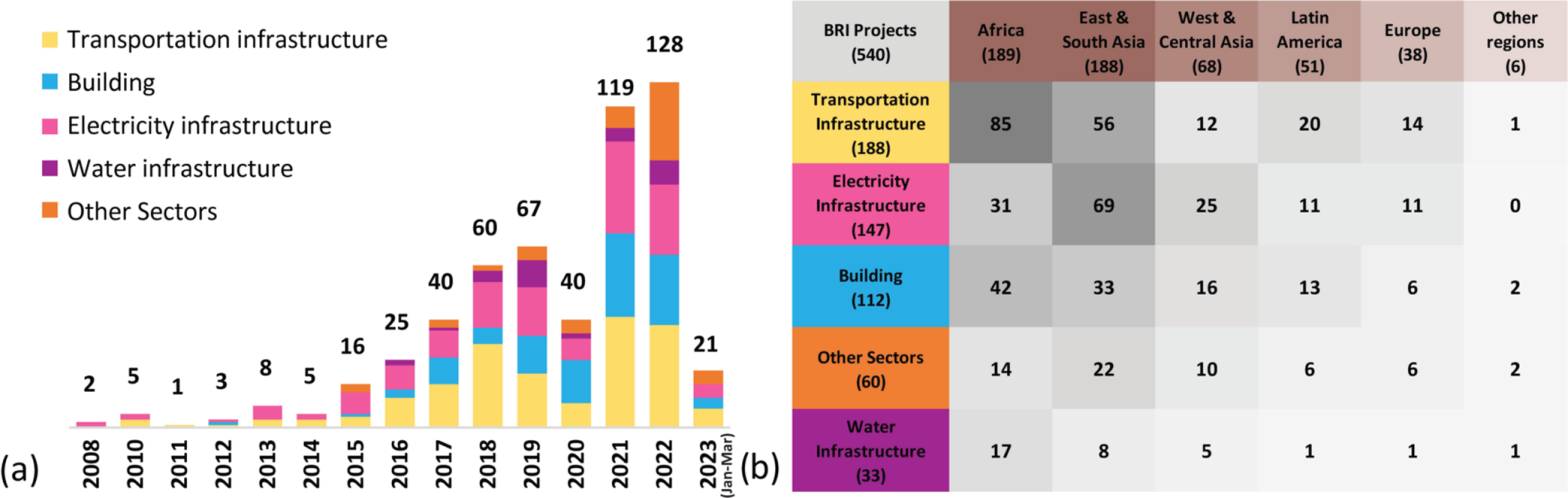
Number of new BRI projects by year, service sector, and region of construction: (a) Number of BRI projects 2008–2023 (March); (b) the distribution of BRI projects across regions and sectors.

Our data covers 540 BRI projects completed or under construction (Figure 2). The number of new projects saw a consistent upward trend from 2013 to 2019. 2020 had a notable decrease in that growth, possibly due to the COVID-19 pandemic, since in 2021 the number of projects increased again, aligning with the growth of previous years. The data for 2023 shows a smaller number of projects because only January to March are covered in this study.

The projects’ distribution across regions and sectors is shown in Figure 2b. 189 projects are located in Africa, accounting for 35% of the total. An almost equal number, 188 projects, are in the East & South Asia region. 68 projects are located in West & Central Asia, making up 13% of the total. Furthermore, there are 51 projects in Latin America, 38 in Europe, and 6 in other regions. In terms of sectors, 188 projects are in the transportation sector, with a little less than half of those located in Africa. Another significant sector is electricity infrastructure, with 147 projects, nearly half of which are in East & South Asia. Furthermore, 112 projects are building construction projects, mainly in Africa and East and South Asia. Water infrastructure accounts for 33 projects. Other projects, such as agricultural facilities, communication cables, and sports facilities, total 36, accounting for less than 12%.

### 3.2 The Material Stocks of BRI Projects

**Figure 3:**
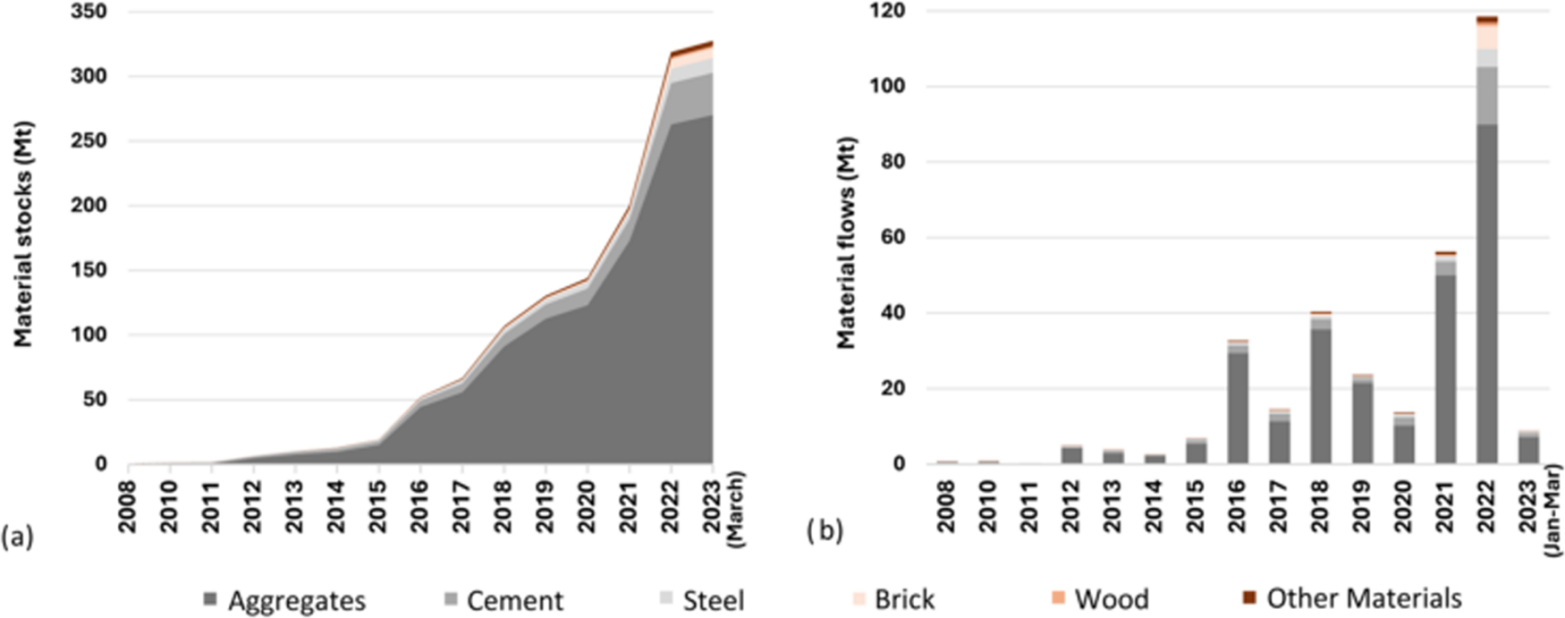
Material stocks and flows of 6 types of construction materials in BRI projects from 2008 to 2023(March): (a) Material stocks; (b) Material flows.

The total stock of construction materials in BRI projects reached 328 Mt (million tons) between 2008 and 2023 (Figure 3a). The initial preofficial BRI years, 2008 to 2011, displayed a moderate and gradual increase, with a more pronounced and steady growth from 2015 onward. By 2018 the material stock reached 107 Mt. In the subsequent five years to 2023, the material stock tripled. Aggregates had the most substantial growth throughout the period, with a significant rise from 2015, culminating at 270 Mt in 2023. Cement follows a similar trend, albeit with a less pronounced acceleration, reaching 33 Mt. The rest of the materials, including steel and bricks, represent a smaller proportion and demonstrated more subtle growth. The average material stock per project is 0.6 Mt, though the biggest projects are buildings in Iraq and railways in Laos with 53.5 and 14.5 Mt, respectively. The material stocks of more than half of the projects are less than 0.1 Mt, and just 12% of the total are over 1 Mt.

Figure 3b displays the annual inflow for the six types of construction materials. In 2008, there were only two electricity infrastructure projects, with no inflow of aggregate, brick, or materials from the “others” category. From 2010 onward, as projects from other sectors surged, a steep rise in the amount of aggregate can be seen. The most significant inflow was in 2022 with 90 Mt of aggregates being added to the stock. Cement’s annual inflow is second-biggest, but even in 2022 it only composed 12% (15 Mt) of total material inflow. The share of steel, bricks, and the rest of the materials remained relatively low.

### 3.3 Transportation Infrastructures Dominate by Mass

**Figure 4:**
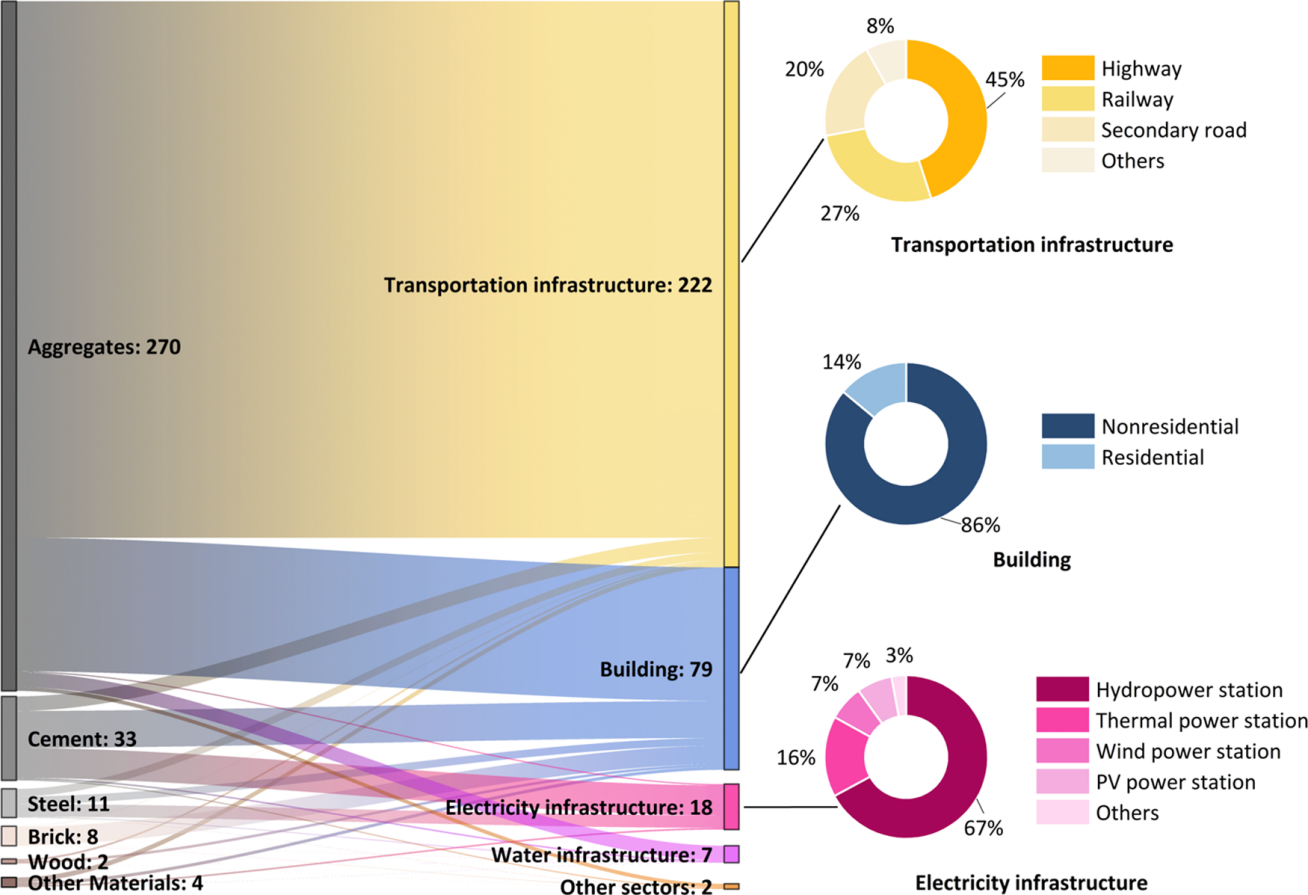
Distributions of the BRI material stocks (Unit: Mt) by 6 construction materials and 5 sectors, with details of subsectors within transportation infrastructure, buildings, and electricity infrastructures.

Figure 4 illustrates the distribution of materials of the BRI projects by sectors. Transportation infrastructure dominates with a material stock of 222 Mt, accounting for 68% of total BRI stocks. Specifically, it consumed 210 Mt of aggregates, which is 78% of the total aggregates use, and 6 Mt of cement (18% of total cement use). Within this sector, roads take up 65% of materials (highway 45% and secondary road 20%), followed by railways. The building sector also has a significant amount of resources, yet a mere third of the transportation sector. Its material stock was 79 Mt of construction materials in 2023, composed of 52 Mt of aggregates, and approximately all the brick stock of 7.3 Mt 86% (67 Mt) of these materials are for nonresidential building construction. Electricity infrastructure contains 18 Mt of construction materials, including one-third of the cement and 40% of the steel. Hydropower stations used the most materials, followed by thermal power stations. Renewable energy power stations, photovoltaics and wind power, each account for 7% of the material stocks in this sector. The rest of the sectors use relatively fewer materials, with water infrastructure using 6.6 Mt and all other sectors using less than 2 Mt.

### 3.4 Asia and Africa Consumed Most of the BRI Construction Materials

**Figure 5:**
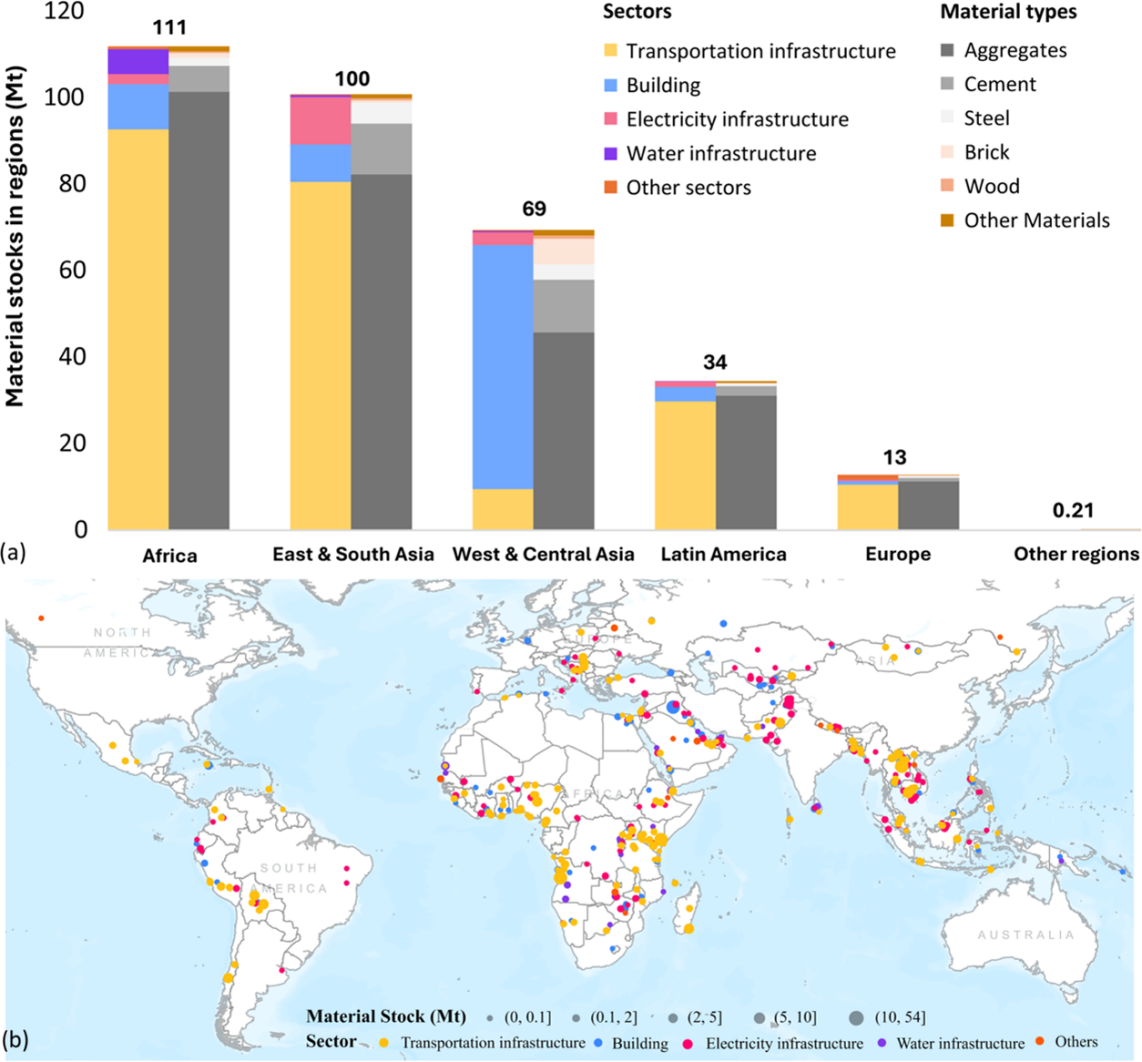
(a) Material stock of BRI projects distribution in regions (compositions of sectors and materials types); (b) detailed mapping of material stocks of 540 BRI projects in 5 sectors.

The regional distribution of the BRI material stock in 2023 is shown in Figure 5a. Most of the stock are in Africa (111 Mt) and East & South Asia (100 Mt), together covering 65% of the whole BRI material stock. The highest proportion of materials in every region is aggregates, accounting for over 90% in Africa and in Latin America. Almost all regions use the most materials in transportation infrastructure projects, except for West & Central Asia, where materials are primarily accumulated in the buildings sector. Hence, the proportion of aggregates is the least in this region compared with others, with a relatively higher proportion of bricks, nearly equaling that used in East & South Asia.

The 540 BRI projects are spread across 97 countries (Figure 5b). The most material-intensive spots are in Southeast Asia, Western Asia, and East Africa. Half of the material stocks are accumulated in just 6 countries: Iraq leads in BRI projects by mass of material stocks (54 Mt), followed by Laos (25 Mt), Cambodia (24 Mt), Kenya (22 Mt), Angola (18 Mt), and Pakistan (16 Mt). The top ten countries account for 63% of total BRI materials.

The material flows for the past decade (SI Figure S2) reveal the growth trends in each region. East & South Asia, Africa, and Central & Western Asia first had construction materials inflows in 2008, 2010, and 2012 respectively, while Latin America and Europe only had inflows after the BRI was formally proposed in 2013. They also showed different patterns of inflow: East & South Asia and Africa have rather steady material inflow after 2015 (except for 2020), while the inflow of other regions fluctuated and most of the inflows do not exceed 10 Mt a year.

## 4 Discussion

### 4.1 The Scale of BRI

**Figure 6:**
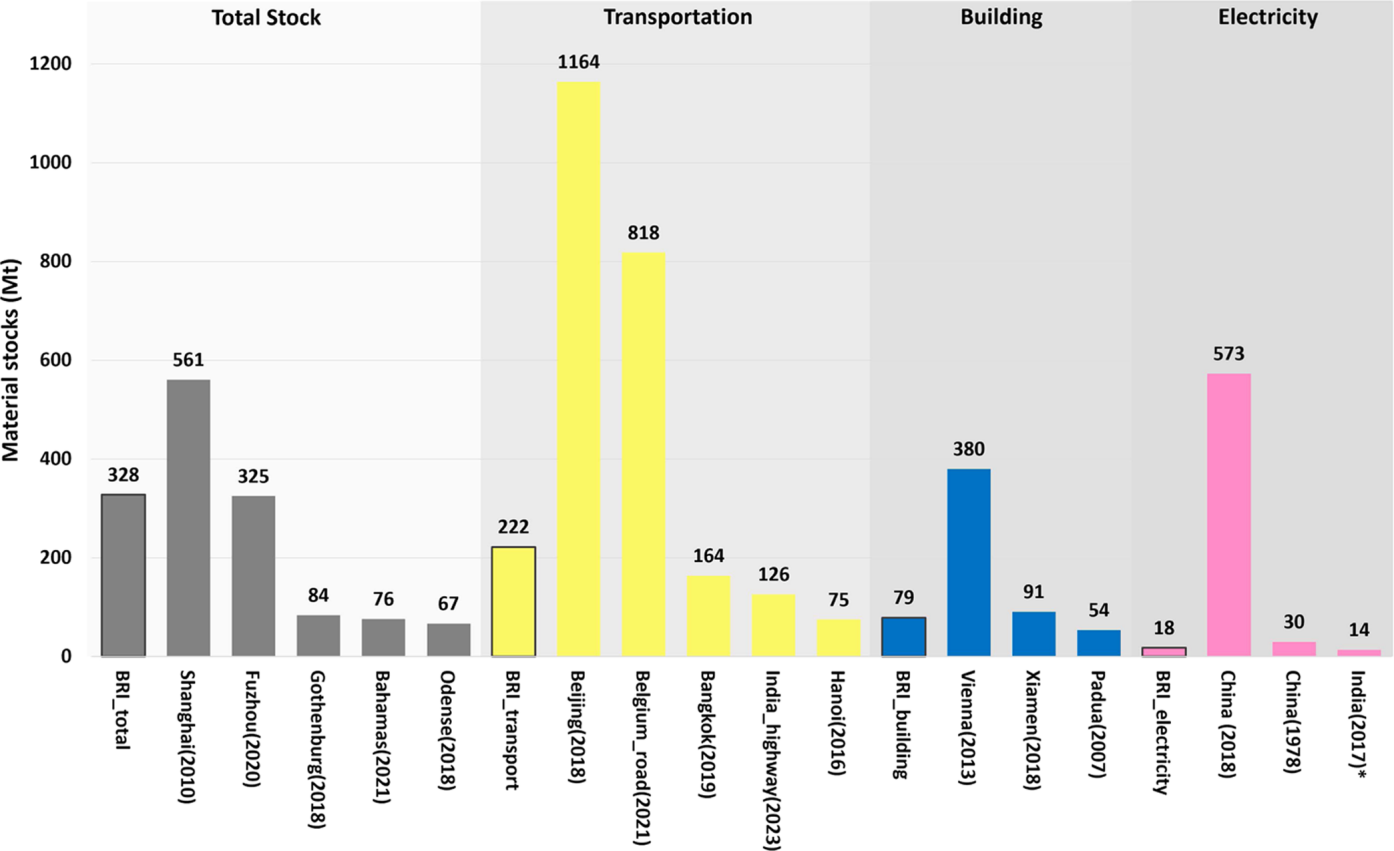
Scale comparison of BRI stock with other studies. The total stock, stocks of transportation infrastructure, buildings, and electricity infrastructure were compared with the according type of stocks. The attached years correspond to the stock years, not to the years when conducting the research or publishing. * Only Steel stocks of power plants in India are showed here.

To give the BRI’s material stocks a sense of scale and check the plausibility of our results, we compare them with other estimates of buildings and infrastructures material stocks (Figure 6). The orders of magnitude are similar to other material stock studies, yet the resources used in BRI projects are considerable. Over the course of merely 15 years, the construction materials of BRI projects have accumulated to 328 million tons, equivalent to over half of the material stocks in Shanghai,^72^ close to the weight of all buildings in Vienna,^73^ and to the total stock of buildings and infrastructures in Fuzhou, China.^74^ The BRI’s total material stock is much larger than the material stock of the Bahamas,^69^ and of some European cities, like Odense in Denmark^75^ and Gothenburg in Sweden.^76^

Sector-specific comparisons show that the BRI transportation infrastructure material stock is larger than that of Hanoi’s and Bangkok’s transport system^67,77^ and is approximately one-fifth of Beijing’s^78^ and one-fourth of Belgium’s.^79^ This is notable, as over 35% of the BRI projects are aimed at developing transportation infrastructure. The average scale of transportation projects is much larger than others (SI Figure S4) and for regions like Africa there are huge infrastructure gaps in transportation.^80^ However, the BRI transport material stock also includes port construction and transportation associated structures, i.e., stations and airport terminals, which are not always included in the compared studies. In just over a decade, the total material stocks of BRI electricity infrastructure surpassed the total stocks of steel in India’s power plants.^81^ It is also over half of that in China in 1980,^71^ prior to China’s period of rapid development, yet is still a small fraction of China’s current electricity infrastructure material stock. The building material stocks in BRI projects are comparable to 90% of the building material stocks of Xiamen, China^82^ and nearly double Padua, Italy.^83^

These comparisons above give a sense of the scale and rapidity of growth of the BRI. The amounts of materials are substantial in terms of resource extraction, production and mobilization across the world. Clearly, the finding that the entire BRI weighs almost the same as Vienna does not make the two interchangeable. While we can compare the weight of the BRI as a single program with the material stocks of cities or states, the composition of material types varies, and the structures of buildings and infrastructure are different. The similarity in mass provides a scale of reference, but does not necessarily translate into comparable service provision. Take one of the BRI sectors, highways, for example: a similar amount of construction materials were used in BRI highways as in all the highways in India.^84^ Yet, conclusions could not be drawn that the BRI highways collectively meet the intercity road transport needs of 1.4 billion people. Nevertheless, the BRI evidently provides benefits in multiple facets, including improved global transportation connectivity and regional development.^85,86^ Weighing the mass of those materials can support a preliminary assessment of certain service provision levels. Measuring the actual service provided by the BRI will be the next challenge, important to assess its societal value.

### 4.2 Implications of BRI Material Stocks

Considering the extensive scale of BRI material stocks, understanding how much and where they serve is significant from both environmental and policy perspectives. Materials accumulated in the built environment were extracted from nature through processing, manufacturing, transport, and construction, raising concerns about these processes’ impacts on sustainable resource use. The materials stocks of the BRI serve as backbones for trade, communication, and other societal services. By mapping the material stocks of individual projects and analyzing them across sectors and regions, we unveil the distribution patterns of material use within the BRI, helping us understand the nexus of material flows, stocks, and service provisioning.^87,88^ As the BRI aligns with development patterns in countries of the Global South, this data could provide reference in reducing infrastructure deficits in the less developed regions.

Broader life-cycle environmental impacts of the accumulated stocks can be assessed based on these material use accounts. This includes especially the embodied impacts from producing these materials, which is generally a significant share of a construction project’s whole life cycle impacts.^89^ The material stocks are also closely linked to their end-use’s operational environmental impacts. By examining subsequent energy needs and carbon emissions during the use phases at multiple levels—individual projects, sectors, regions, and the entirety of the BRI—our work contributes to the possibility to assess the BRI’s sustainability from a holistic view.

Furthermore, such information is important to identify future material demands for maintaining and expanding current BRI projects, and hence potential opportunities to incorporate recycled materials, employ more sustainable options, and improve material efficiency to reduce reliance on virgin raw materials, thereby avoiding the overexploitation of natural sources. In addition, anticipating future waste flows from these projects can also help plan for their further treatments and potential use as secondary materials. Geoinformation on the projects’ material inflows and future outflows will facilitate the recycling and reuse process, saving time on matching the demand and supply and reducing costs and impacts associated with transport. Understanding the material inflows and managing the outflows can foster sustainable resource use in infrastructure growth through the BRI.^75,90,91^

### 4.3 Data Quality and Uncertainties

This is the first attempt to map and account for the total mass of materials of an international initiative like the BRI, whose definitions are somewhat vague and shifting over time even in official Chinese documents. This attempt enables us to identify some challenges concerning the compilation and estimation of the database, as well as uncertainties of our results arising from both accounting units and material intensities.

#### 4.3.1 Omitted Stocks

Material stocks in some BRI project types such as leisure facilities, mines, vehicles, and oil fields could not be estimated. While we documented them in our database, we do not have sufficient information from official sources, and neither geoinformation nor reliable assumptions can be made to account for their units and subsequent calculation of their masses. For full coverage and potential future estimation once data becomes available, we mark them as “omitted in project unit” (SI. Figure S1), and their material stocks are therefore also unaccounted.

#### 4.3.2 Possibly Omitted Projects

The official BRI portal Web site began to document weekly reports from 2021, thus the information on projects in our database for the recent 3 years is more complete than those from before. In addition, the BRI portal did not announce whether these reports include all projects. Therefore, there could be projects that were finalized before 2021 and the weekly reports did not mention them, leading to complete omission from our data.

#### 4.3.3 Uncertainties of the Material Inflow Years

The material inflows of each year are based on the simplified assumption that all materials used to construct a project flowed into the BRI at the beginning year, without considering the construction length or year of completion, which are often not available. This could lead to a mismatch between the year and its actual BRI stock accumulation. If data on the length and dynamics of construction times for the various construction projects becomes available, this assumption could be relaxed. While this mismatch may be negligible for the overall trends and aggregated scales, one manifestation of this is that our results show that material inflows started in 2008, which requires further confirmation. For example, the construction of Neelum-Jhelum hydropower project in Pakistan started in 2008. Both the hosting country and China declared it as a BRI project, so we include it in our database and its entire material flow is documented in 2008 as construction of the power station began.

#### 4.3.4 Material Outflows are not Accounted

We assume there is no outflow from all these newly built infrastructures, due to lack of usable data to show otherwise. However, there is bound to be some material outflow. For example, there are materials excavated from the site, on-site construction waste, and so on. For now, we have no way to estimate the amount of material loss or accumulation. Furthermore, as time progresses, eventually the oldest projects may reach their end of life and be demolished, but we did not identify any information that could suggest that this has already occurred.

#### 4.3.5 Material Intensities

Differences in the same type of project can be expected, due to local standards and conditions. However, in this study we treat them as equivalent structure types and use one set of archetypal material intensities to account for them. The use of archetypes is a well-known and yet unresolved challenge in bottom-up material stocks assessments.^42,43,92,93^ Besides that, our estimate focused on the primary construction materials, and some critical materials, e.g., Indium in solar cells and Neodymium in offshore wind turbines, are not accounted in our database so far. The quantity of these materials will not affect the magnitude of the entire BRI weight, but the scarcity of these critical materials has been a focal point of concern.^94−96^ Furthermore, we separate concrete into its constituent cement and aggregate for consistency. We assumed a fixed ratio of these materials, which could be improved and further differentiated if information on concrete characteristics becomes available in the future.

Uncertainties could arise from the choice of MIs. Chinese standards are prioritized as most BRI projects were designed by Chinese firms, and MIs for developing regions align with our scope given that most of BRI projects are located in such areas. Therefore, a generalized set of single-value MIs per material for each end-use is adopted to represent the archetypes and estimate the corresponding stock. MIs might vary by region, but such variability is not yet captured in the current state-of-the-art methods or the available literature. Under such conditions, changing the MIs to other single values from a different source lead to an equivalent percent-change in the results. Furthermore, single-value MIs cannot capture the inherent variability of different construction projects, yet such variations would have diminishing impacts on the overall aggregated findings. Combined with potential uncertainties arising from the challenges of determining the accounting units’ sizes, our focus is on highlighting the trends and patterns in the BRI material stocks rather than emphasizing absolute numerical assessment. This strengthens the call for further investigations and quantifications of bottom-up material stocks that could provide additional validation and refinement of richer MIs from the perspective of locations, more detailed structure types, construction periods, etc., which would then lead to a more nuanced data set. Nevertheless, the accounting unit of each project is recorded well. It will not be difficult to update our material stock database once better versions of MI ranges become available.

### 4.4 Outlook for Further Research

To the best of our knowledge, this is the first estimation of the materials used specifically in the BRI’s development projects. We provide a first step toward a sustainability analysis by quantifying these stocks. The results show material use in total and per material, for every project, sector, and region individually. The detailed mapping of the material distribution can be a starting point for the analysis of future material demand for maintenance, and of the expected waste flows related to maintenance and obsolescence. It can also provide a reference for identifying infrastructure gaps in underdeveloped countries. This database will continue getting updated with new projects and revised estimates as new information becomes available, with the aim to make it publicly accessible (the data compiled so far is available upon request). Further research can assess the environmental impacts related to the BRI construction, and quantify, for example, energy consumption, ecosystem service changes, and carbon emissions from this large infrastructure program. Making a connection to global service provision, the stock-flow-service nexus can also be explored through this data.

## Supporting Information Available

Four tables on countries and regions involved in BRI, categories for sectors/project types/structure types, total size for each structure type and original MIs for bridge, airport runways, and port platforms; 3 figures on method to decide accounting unit, material flows in regions, and average/median scale for BRI projects (PDF)
