# Supplementary material for: A Comprehensive Accounting of Construction Materials in Belt and Road Initiative Projects
Source: Environ Sci Technol. 2024 Aug 20;58(35):15575–86. doi: 10.1021/acs.est.4c04142 (PMC11375774; doi:10.1021/acs.est.4c04142)
Supplement: Supplementary file 1 — es4c04142_si_001.pdf [file es4c04142_si_001.pdf]

Supporting Information

A Comprehensive Accounting of Construction Materials in Belt and Road Initiative Projects

Lingli Hou [a,*] , *Tomer Fishman* [a], *Ranran Wang* [a], *Asaf Tzachor* [b,c], *Heming Wang* [d,e], *Peng Wang* [f,g], *Wei-Qiang Chen* [f,g], *Ester van der Voet* [a]

[a] Institute of Environmental Sciences (CML), Leiden University, Leiden 2333CC, The Netherlands

[b] School of Sustainability, Reichman University, Herzliya 4610101, Israel

[c] Centre for the Study of Existential Risk (CSER), University of Cambridge, Cambridge, UK

[d] State Environmental Protection Key Laboratory of Eco-Industry, Northeastern University, Shenyang 110819, China.

[e] Commonwealth Scientific and Industrial Research Organisation (CSIRO), Canberra, ACT 2601, Australia

[f] Key Lab of Urban Environment and Health, Institute of Urban Environment, Chinese Academy of Sciences, Xiamen, Fujian 361021, China

[g] University of Chinese Academy of Sciences, Beijing, 100049, China

[*] Email: l.hou@cml.leidenuniv.nl

Contents

Number of pages: 9

Number of tables: 4

Number of figures: 3

Table S1. Country and region for BRI projects S2

Table S2. Sector-project type-structure type of BRI projects S4

Table S3. Total size for each structure in BRI projects S5

Table S4. Material intensities of bridges, airport runways, and port platforms in BRI S5

Figure S1. Flowchart of deciding accounting unit of BRI projects S6

Figure S2. BRI project material flows in 5 regions (Unit: Mt) S7

Figure S3. Average & median scale of BRI projects in sectors and regions S8

Table S1. Country and region for BRI projects

Region	Country	Region	Country
**Africa**	Algeria	**East & South Asia**	Singapore
**Africa**	Angola	**East & South Asia**	Sri Lanka
**Africa**	Benin	**East & South Asia**	Thailand
**Africa**	Botswana	**East & South Asia**	Vietnam
**Africa**	Burkina Faso	**Europe**	Belarus
**Africa**	Burundi	**Europe**	Belgium
**Africa**	Cameroon	**Europe**	Bosnia and Herzegovina
**Africa**	Central African Republic	**Europe**	Croatia
**Africa**	Comoros	**Europe**	Hungary
**Africa**	Côte d'Ivoire	**Europe**	Italy
**Africa**	Djibouti	**Europe**	Moldova
**Africa**	DRC	**Europe**	Montenegro
**Africa**	Egypt	**Europe**	Norway
**Africa**	Ethiopia	**Europe**	Poland
**Africa**	Ghana	**Europe**	Portugal
**Africa**	Guinea	**Europe**	Russia
**Africa**	Kenya	**Europe**	Serbia
**Africa**	Lesotho	**Latin America**	Argentina
**Africa**	Madagascar	**Latin America**	Bolivia
**Africa**	Malawi	**Latin America**	Brazil
**Africa**	Mali	**Latin America**	Chile
**Africa**	Mauritania	**Latin America**	Colombia
**Africa**	Mozambique	**Latin America**	Costa Rica
**Africa**	Namibia	**Latin America**	Dominica
**Africa**	Niger	**Latin America**	Ecuador
**Africa**	Nigeria	**Latin America**	Grenada
**Africa**	Republic of the Congo	**Latin America**	Guyana
**Africa**	Rwanda	**Latin America**	Jamaica
**Africa**	Senegal	**Latin America**	Mexico
**Africa**	Sierra Leone	**Latin America**	Peru
**Africa**	Tanzania	**Others**	Canada
**Africa**	Tunisia	**Others**	Papua New Guinea
**Africa**	Uganda	**Others**	Samoa
**Africa**	Zambia	**Others**	Solomon
**Africa**	Zimbabwe	**West & Central Asia**	Bahrain
**East & South Asia**	Afghanistan	**West & Central Asia**	Georgia
**East & South Asia**	Bangladesh	**West & Central Asia**	Iraq
**East & South Asia**	Cambodia	**West & Central Asia**	Israel
**East & South Asia**	East Timor	**West & Central Asia**	Jordan
**East & South Asia**	Indonesia	**West & Central Asia**	Kazakhstan
**East & South Asia**	Laos	**West & Central Asia**	Kuwait
**East & South Asia**	Malaysia	**West & Central Asia**	Kyrgyzstan
**East & South Asia**	Maldives	**West & Central Asia**	Oman
**East & South Asia**	Mongolia	**West & Central Asia**	Qatar

**East & South Asia**	Myanmar	**West & Central Asia**	Saudi Arabia
**East & South Asia**	Nepal	**West & Central Asia**	Tajikistan
**East & South Asia**	Pakistan	**West & Central Asia**	Turkey
**East & South Asia**	Philippines	**West & Central Asia**	United Arab Emirates
		**West & Central Asia**	Uzbekistan

Table S2. Sector-project type-structure type of BRI projects

Sector	Structure type	Projects type
**Transportation infrastructure**	Railway	Railway
	Subway	Subway
	Light rail	Light rail
	Highway	Highway
	Secondary road	Secondary road
	Bridge	Bridge
	Airport runway	Airport
	Nonresidential building	Railway station*
	Nonresidential building	Airport terminal*
	Port platform	Port
**Building**	Residential building	Residential building
	Nonresidential building	Office
		Hospital
		Hotel
		Plant
		School
		Warehouse
		Lab
**Electricity infrastructure**	Hydropower station	Hydropower station
	Thermal power station	Thermal power station
	Wind power station	Wind power station
	PV power station	PV power station
	Nuclear power station	Nuclear power station
	Transmission	Transmission
**Water infrastructure**	Water supply pipeline	Tap water pipeline
	Sewer pipeline	Sewer pipeline
	Nonresidential building	Water plant*

Note: *The structure is accounted as nonresidential buildings, however, the material stock is accumulated in the sector according to the service it provided. Other sectors are not listed here, but most of them are accounted, if accounting unit is available, as nonresidential buildings or roads, e.g. industrial park (including buildings and roads) and gas processing plant (non-residential).

Table S3. Total size for each structure in BRI projects

Project Type	Accounting Unit	Unit	Project Type	Accounting Unit	Unit
**Railway**	10,430	km	**Nonresidential**	32,150,275	m^2
**Subway**	166	km	**Thermal Power Station**	19,192	MW
**Light rail**	105	km	**Hydropower Station**	26,493	MW
**Highway**	51,713,745	m^2	**Wind Power Station**	4,089	MW
**Secondary Road**	33,198,452	m^2	**Photovoltaic Power Station**	9,535	MW
**Bridge**	1,428,664	m^2	**Nuclear Power Station**	1,100	MW
**Airport Runways**	2,878,220	m^2	**Transmission**	5,779	km
**Port Platforms**	687,614	m^2	**Tap Water Pipeline**	1,570	km
**Residential**	7,344,998	m^2	**Sewer Pipeline**	23	km

(km: kilometers; m^2: square meters; MW: Megawatt.)

Table S4. Material intensities of bridges, airport runways, and port platforms in BRI

Structure Type	Unit	Steel	Wood	Concrete	Aggregates	Asphalt	Reference
**Bridge**	t/m^2	0.263	0.518	2.971		0.146	1
**Airport Runways**	t/m^2			0.305	0.427	0.234	2
**Port Platforms**	t/m^2			0.366	0.64		

Note: Concrete is separated into cement/aggregate/water with the ratio of 13%/80%/7%.[3,4]

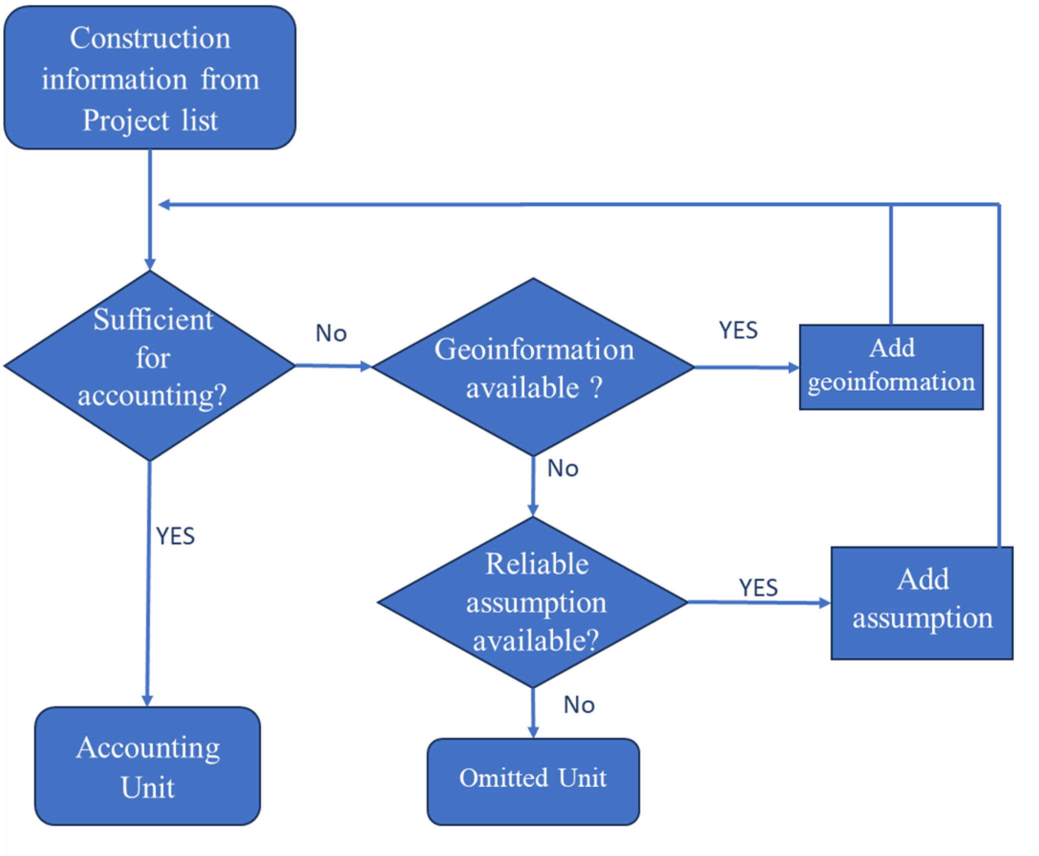

Figure S1. Flowchart of deciding accounting unit of BRI projects

Note: To identify the project accounting unit, firstly we evaluate if the information from the project list is sufficient to determine it. If yes, which means the descriptions indicate enough data to do the accounting, we take the numbers directly from them. If no, we will consider if there are geoinformation of these projects. If yes, geoinformation, for example google map or site pictures of the projects, is available, we integrate it with our basic data from description to produce the accounting unit. If no, there is no geoinformation or site photos for us to make a model to calculate, we will consider if there are reliable assumptions for the projects. Reliable assumptions include taking one project as benchmark to assume the accounting unit of other projects in the same type yet lacking information and assume the size of one project from other studies or average size. For some project we cannot decide a benchmark project or no other ways to make assumptions, we treat them as omitted size and then they became omitted stocks.

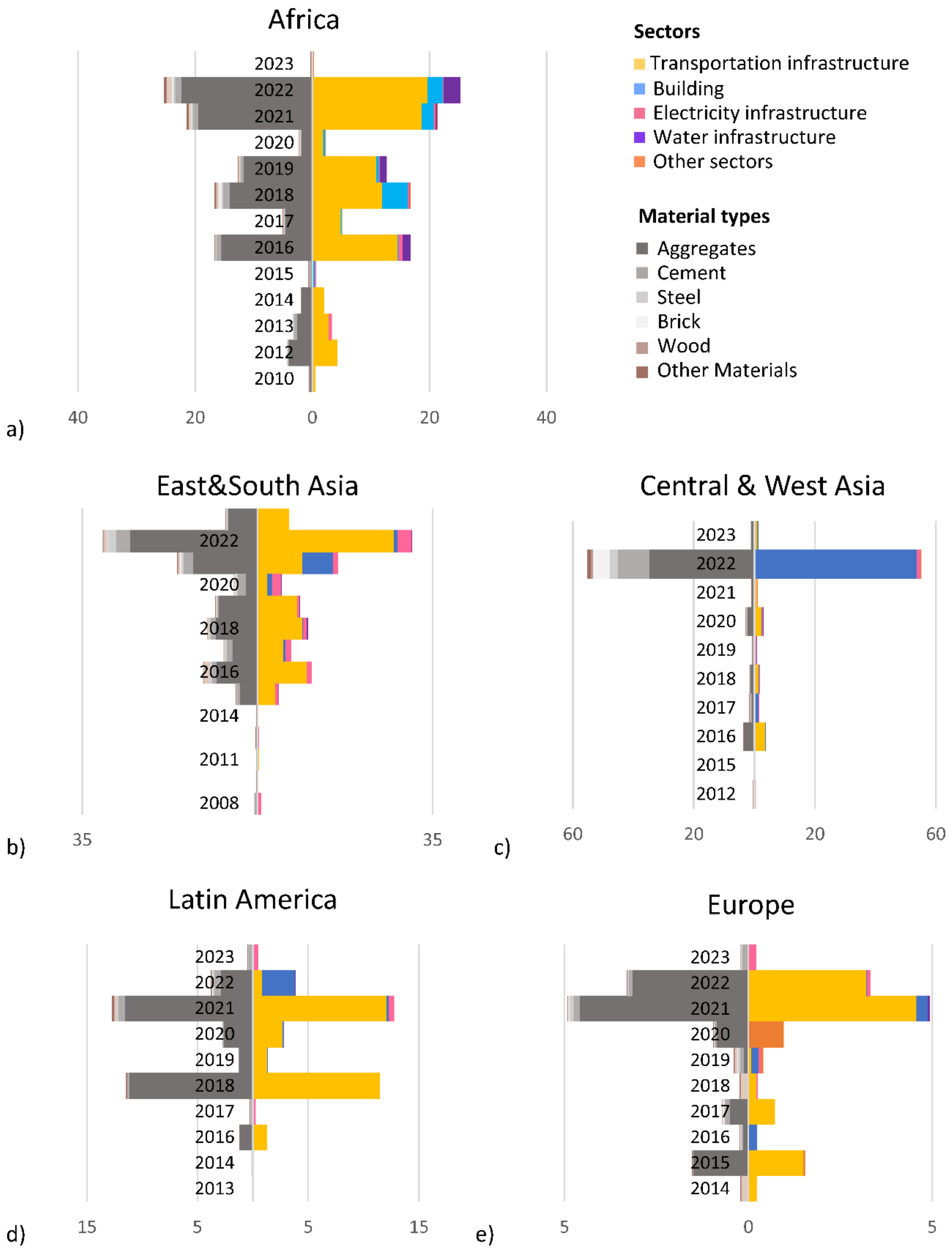

Figure S2. BRI project material flows in 5 regions (Unit: Mt)

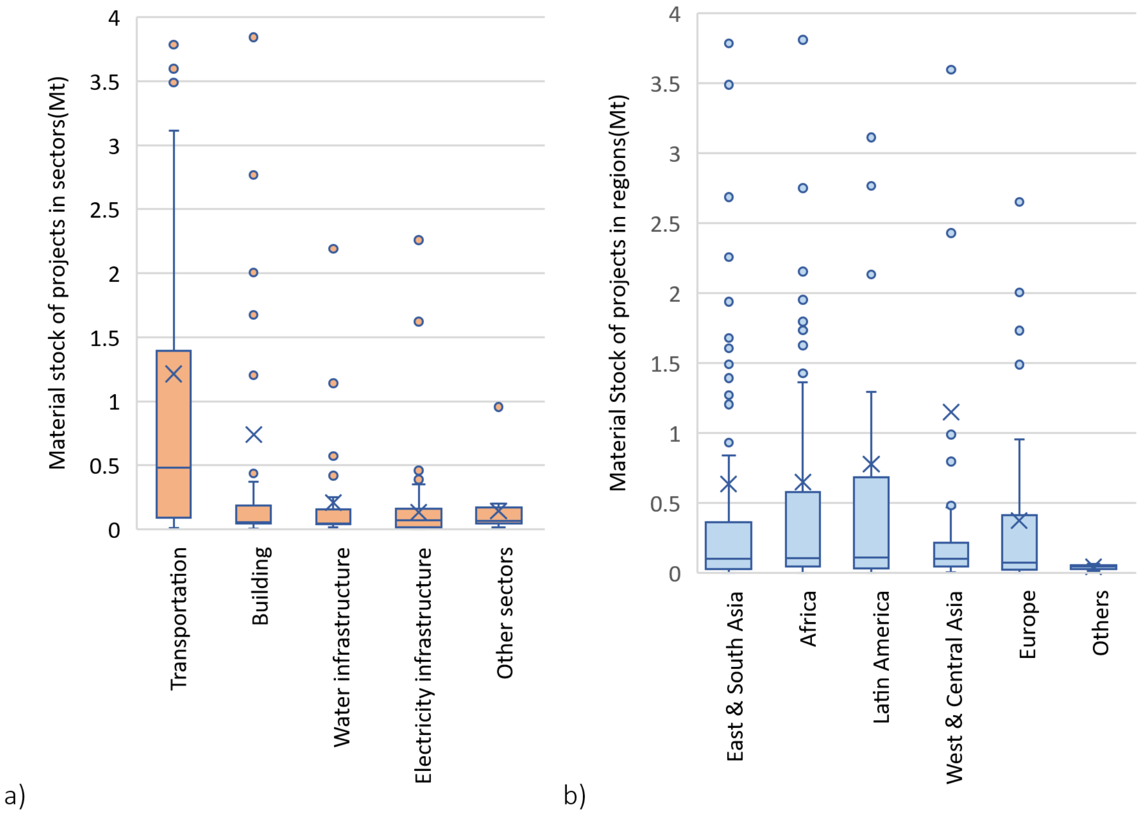

Figure S3. Average & median scale of BRI projects in sectors and regions

Reference

(1) Hammervold, J.; Reenaas, M.; Brattebø, H. Environmental Life Cycle Assessment of Bridges. *J. Bridge Eng.* **2013**, *18* (2), 153–161. https://doi.org/10.1061/(ASCE)BE.1943-5592.0000328.
(2) Martin del Campo, F.; Singh, S. J.; Fishman, T.; Thomas, A.; Drescher, M. The Bahamas at Risk: Material Stocks, Sea-Level Rise, and the Implications for Development. *J. Ind. Ecol.* **2023**, *27* (4), 1165–1183. https://doi.org/10.1111/jiec.13402.
(3) Turk, J.; Cotič, Z.; Mladenovič, A.; Šajna, A. Environmental Evaluation of Green Concretes versus Conventional Concrete by Means of LCA. *Waste Manag.* **2015**, *45*, 194–205. https://doi.org/10.1016/j.wasman.2015.06.035.
(4) Watari, T.; Cao, Z.; Hata, S.; Nansai, K. Efficient Use of Cement and Concrete to Reduce Reliance on Supply-Side Technologies for Net-Zero Emissions. *Nat. Commun.* **2022**, *13* (1), 4158. https://doi.org/10.1038/s41467-022-31806-2.
